# The Association Between Economic Status and Religious Identity With Oral Health Disparities and Inequalities Around the World

**DOI:** 10.7759/cureus.51917

**Published:** 2024-01-08

**Authors:** Farooq Ahmad Chaudhary, Basaruddin Ahmad, Bilal Arjumand, Hamad Mohammad Alharkan

**Affiliations:** 1 Department of Community Dentistry, School of Dentistry, Shaheed Zulfiqar Ali Bhutto Medical University, Islamabad, PAK; 2 Department of Dental Public Health, School of Dental Sciences, Universiti Sains Malaysia, Kota Bharu, MYS; 3 Department of Conservative Dental Sciences and Endodontics, College of Dentistry, Qassim University, Buraydah, SAU

**Keywords:** socioeconomic status (ses), religion, periodontal status, disparity, oral health

## Abstract

Introduction: The inequalities in oral health remain one of the current issues in the global public health agenda. The number of studies investigating health disparity by religious identity is limited and there is currently no such report relating to oral health. Similarly, there is compelling evidence for oral health disparities between socioeconomic statuses, education levels, and ethnic groups. This ecological study aimed to explore the disparity in oral health-related outcomes between Muslim and non-Muslim countries and country income status.

Methods: Publicly available data related to oral health measures, country income status, and membership in the Organization of Islamic countries were used. Five oral health-related measures were examined: caries experience (decayed, missing, and filled teeth (DMFT)), percentage of the population with no periodontal disease, and disability-adjusted life years (DALY) attributed to oral conditions, and mouth and oropharynx cancer. One-way analysis of variance (ANOVA) and Kruskal-Wallis tests were used to compare the oral health parameters by country income status and simple linear regression was used to compare the parameters between the non-member countries (n-MC) and member countries (MC). For the significant parameters, adjusted coefficients were obtained using multiple linear regression.

Results: From 170 countries included, 53 (31%) were MC and 117 (69%) were n-MC. Analysis showed that the mean DMFT in adults aged 35-44 years was significantly higher in the n-MC compared to MC after adjusting for country income status (p<0.05) but the latter was the stronger explanatory predictor of the outcome. The strength of the effect of country membership classification (standardized coefficient β: DMFT_35-44-year-old_ = -0.16) was smaller than country income status (β = -0.60) in the multiple regression.

Conclusion: There is significant but weak evidence from the available data to support the claim that economic status and religion contribute to oral health disparity.

## Introduction

There is compelling evidence for oral health disparities between socioeconomic statuses, education levels, and ethnic groups, and the particular address in the Lancet Series suggests that inequalities in oral health remain one of the current issues in the global public health agenda [1-5]. In the last few decades, several health indicators of the population showed an improvement in many countries, but health inequalities have become one of the major health problems in many countries [1,3,5]. People in the lower socioeconomic group, less educated, and of marginalized and socially excluded groups in societies have poorer oral health compared to their counterparts [1,5].

There was a suggestion that discriminating minority groups based on their religious belief can have negative consequences on the quality and delivery of care and result in health disparities [6]. Religion plays an important and significant part in sculpting social behavior and role in human societies. Every religion and culture has its own customs which may have a significant influence on health and oral health. To date, the focus of research is on the impact of generalized religiosity and the importance of following and attending religious services, and to some extent on the specific religious practices on health outcomes without much attention to the way religion shapes the health behaviors of individuals. One ecological study compared several health outcomes between the Muslim and non-Muslim majority countries and found that the former had a significantly greater mortality rate in children < 5 years old and a lower fertility rate in women but no difference in life expectancy and adult mortality rate [7]. The authors claimed that religion plays an equitable role as other determinants such as wealth, education, and infrastructure in a complex causal pathway to influence the health gradients between the populations. However, the number of studies investigating health disparity by religious identity is limited and there is currently no such report relating to oral health [6,7]. Thus, this study was undertaken to explore whether there is a disparity in oral health-related outcomes between Muslim and non-Muslim countries and country income status using secondary data.

## Materials and methods

This ecological study included all countries worldwide [8] that have oral health data relating to caries experience, periodontal disease, and Disability-Adjusted Life Year (DALY) (per 100,000 population) due to oral conditions. Small countries with a population of less than 100,000 were excluded because they lacked socio-economic data.

The study was approved by the Ethical Review Board of the School of Dentistry, Shaheed Zulfiqar Ali Bhutto Medical University, Islamabad, Pakistan (Ref. No. SOD/ERB/2021/122). This study used secondary data from reliable sources in the public domain [9,10]. Oral health outcome data, the DMFT (decay, missing, filled teeth) index of 12-years-old (DMFT12yo) and 35-44-year-old (DMFT35-44yo), percentage of the population with healthy periodontium in 35-44-year-old (HP35-44yo), DALY due to oral conditions, and DALY due to mouth and oropharynx cancer, were obtained from the World Health Organization (WHO), the Global Health Observatory, oral health data portal [9-11] which obtains, maintains, and updates the information from various sources including national survey reports, and Oral Health database CAPP (Country/Area Profile Project) maintained by the WHO Collaborating Centre, Malmö University, Sweden, national health bulletins and personal communication [9-11]. The DMFT measures the severity of dental caries experience. It is assessed using clinical examination, in which each sound tooth in the oral cavity is scored as ‘0’ and that with an active caries lesion, a restoration or missing due to extraction is scored as ‘1’. The total score ranges from 0 to 32 and an increasing value corresponds to a more severe oral condition. Periodontal status is measured using the Community Periodontal Index which ranges from 0 (healthy periodontium), 1 (gingival bleeding), 2 (calculus), 3 (periodontal pocket 4-5mm), and 4 (periodontal pocket >6mm) [12]. The percentage of HP35-44yo in this study refers to the CPI = 0. The information on DALY was obtained from the WHO Global Health Observatory Data Repository [11,13]. It measures the burden of oral disease and is interpreted as the number of potentially productive years lost due to oral health-related disability or early death due to oral condition. Two measures were used: the age-standardized DALY due to oral condition (DALYoral), and mouth and oropharynx cancer (DALYcancer).

The countries were classified as member countries (MC) and non-member countries (n-MC) of the Organization of Islamic Cooperation (OIC) [14] based on their membership in the organization irrespective of the number of Muslim populations in the country. The Gross National Income per capita (GNI) (in USD currency, divided by the midyear population) in 2018 was obtained from the World Bank website [15] and it was used to classify the countries by income status: low-income (≤ USD 1,035) (LIC), lower-middle-income (USD 1,036 to 4,085) (LMIC), upper-middle-income (USD 4,086 to 12,615) (UMIC) and high-income countries (≥ USD 12,616) (HIC) [16] (see Table 4 in Appendices for details of the GNI per capita income groups countries). Data from the different sources were combined and matched by the country name.

Descriptive analysis was carried out to describe all variables by the n-MC/MC groups. One-way analysis of variance (ANOVA) and Kruskal-Wallis tests were used to compare the oral health parameters by country income status. Analysis to compare the oral health outcomes between the n-MC and MC was carried out using the simple linear regression and for the significant parameters, adjusted coefficients were obtained using the multiple linear regression. The oral health outcome measures were used as the dependent variable. The significance level was set at 5%. All analyses were carried out using the Statistical Package for the Social Sciences (IBM SPSS Statistics for Windows, IBM Corp., Version 24.0, Armonk, NY).

## Results

A total of 170 countries were included in this study, 117 (69%) were n-MC and 53 (31%) MC (Table 1) (see Table 5 in Appendices for details of the countries). Twenty-five countries were excluded from the study because the relevant oral health data was not available. The total number of parameters per country varied because not all data were available in the database. The year of publication of the oral health-related data ranged from 1996 to 2021. There were more HIC and fewer LIC in the n-MC compared to the MC (Table 1).

**Table 1:** The distribution of the number of countries included in the study by oral health parameters and country classification. GNI: Gross National Income; DMFT: Decay, missing, filled teeth; DALY: Disability-Adjusted Life Year

Variables	Non-Muslim Countries N (%)	Muslim Countries N (%)
All countries (n=170)	117 (69%)	53 (31%)
GNI		
High (n=50)	43 (36.8)	7 (13.2)
Upper middle (n=46)	31 (26.5)	15 (28.3)
Lower middle (n=41)	26 (22.2)	15 (28.3)
Lower (n=33)	17 (14.5)	16 (30.2)
DMFT		
12 years (n=166)	117 (70.5)	49 (29.5)
35-44 years (n=110)	76 (69.1)	34 (30.9)
Periodontal disease free in 35-44 years (n=84)	60 (71.4)	24 (28.6)
DALY: Oral conditions (n=170)	117 (68.8)	53 (31.2)
DALY: Mouth and oropharynx cancer (n=168)	115 (68.5)	53 (31.5)
Per Capita Consumption of Sugar (kg) (n=156)	107 (68.6)	49 (31.4)

Table 2 summarizes the oral health-related parameters by the OIC membership classification and country income status. There were associations between country income status and all oral health-related parameters except the percentage of the population with healthy periodontium (p<0.01) (Table 2).

**Table 2:** The summary of oral health-related parameters by country classification and income status. ^a ^Percentage with no periodontal disease, ^b^ Disability-Adjusted Life Year (DALY), ^c ^Estimated DALY due to oral conditions, age-standardized, per 100,000 population, ^d^ Estimated DALY due to mouth and oropharynx cancer, age-standardized, per 100,000 population, ^e^ high vs upper middle, ^f^ high vs lower middle, ^g^ high vs lower ^h^ upper middle vs lower middle, ^i ^upper middle vs lower, ^j^ lower middle vs lower, ^k ^one-way ANOVA, post hoc: Games Howell, ^l ^one-way ANOVA, post hoc: LSD, ^m ^Kruskall-Wallis, pairwise: Bonferroni, * Interquartile range DMFT: Decay, missing, filled teeth

	Country Income Status														
	High Income			Upper Middle			Lower Middle			Lower			All		P-value
DMFT 12 years															
	Mean	SD	n	Mean	SD	n	Mean	SD	n	Mean	SD	n	Mean	SD	
Non-Muslim countries	1.7	1.02	43	2.4	1.44	31	2.2	1.24	26	1.1	1.03	17	1.9	1.27	0.001^i,k^
Muslim countries	2.7	1.96	7	2.6	1.08	14	1.4	0.78	15	1.5	0.91	13	1.9	1.23	
DMFT 35-44 years															
	Mean	SD	n	Mean	SD	n	Mean	SD	n	Mean	SD	n	Mean	SD	
Non-Muslim countries	14.2	3.6	31	11.6	4.3	15	7.9	4.0	18	5.1	3.0	12	10.8	5.0	0.004^e,f,g,h,I,j,l^
Muslim countries	7.4	1.9	5	10.5	4.0	11	7.6	3.1	10	5.0	2.6	8	7.7	3.5	
Percentage with healthy Periodontium (35-44-year-old^a^)															
	%	SD	n	%	SD	n	%	SD	n	%	SD	n	%	SD	
Non-Muslim countries	6.6	6.36	28	2.3	3.47	11	4.5	8.35	12	8.3	11.1	9	5.7	7.38	0.1
Muslim countries	20	-	1	3.4	4.00	7	2.6	3.28	8	5.5	11.9	8	4.5	7.98	
DALYs^b^: Oral conditions^c^															
	Median	IQR*	n	Median	IQR*	n	Median	IQR*	n	Median	IQR*	n	Median	IQR*	
Non-Muslim countries	83.0	28.0	43	170.0	123	31	142.5	98.0	26	99	21.0	17	111.0	119.0	<0.001^e,f,h,m^
Muslim countries	225.0	1	7	191	115	15	170.0	93.0	15	100.5	59.0	16	170.0	109.0	
DALYs^b^: Mouth and oropharynx cancer^d^															
	Median	IQR	n	Median	IQR	n	Median	IQR	n	Median	IQR	n	Median	IQR	
Non-Muslim countries	39.5	32	42	40.0	51.0	30	53.5	52.0	26	63.0	43.0	17	50.0	43.0	<0.001^f,g,m^
Muslim countries	23.0	49.0	7	48.0	43.0	15	67.0	36.0	15	60.5	35.0	16	60.0	45.0	

The simple linear regression showed that that the mean DMFT35-44-year-old and sugar consumption was significantly lower in the MC than n-MC (Table 3). Only the association between DMFT35-44-year-old and country membership classification remained significant after adjusting for country income status (mean difference = -1.6, p< 0.05). The strength of the effect of country membership classification (standardized coefficient β: DMFT35-44-year-old = -0.16) was smaller than country income status (β = -0.60) in the multiple regression.

**Table 3:** Simple and adjusted* linear regression coefficients for the association between oral health-related parameters and country classification. * adjusted for GNI; SLR: Simple linear regression; b: regression coefficient; DMFT: Decay, missing, filled teeth; DALY: Disability-Adjusted Life Year; GNI: Gross National Income

	SLR b (95% Cl)	p	Adjusted* b (95% Cl) β	P-value
DMFT 12 years old children	0.03 (-0.39, 0.45)	0.9	-	
DMFT 35-44 years old adults	-2.82 (-4.74, -0.91)	0.004	-1.58 (-3.13, -0.032) (0.05)	0.046
Periodontal disease-free in the age group 35-44	-1.13 (-4.75, 2.50)	0.5	-	
DALY by Oral conditions	17.58 (-1.38, 36.54)	0.1	-	
DALY: Mouth and oropharynx cancer	4.56 (-10.18, 19.30)	0.5	-	
Sugar Consumption per capita (kg)	-7.826 (-13.10, 2.55)	0.004	-	

## Discussion

This study examined several oral health indices gathered from various reliable sources for evidence of health disparities between non-Muslim and Muslim countries and country income status. The analysis found that caries experience in adults aged 35-44 years old is lower in the MC compared to the n-MC after accounting for country income status. Country income status also has a stronger effect on the association. The finding of this study is not directly comparable to other reports due to its uniqueness. Nevertheless, a report on ethnic inequalities in dental caries among 16 to 65-year-old adults in East London showed that the Asian community, which the majority consisted of Muslim Pakistani and Bangladeshi, have lower DMFT and, missing and filled teeth than White British [17] and in another report, they are shown to have worse periodontal status compared to the White British [3].

The earlier report on health disparities which showed that Muslim countries have poorer health than non-Muslim countries based on the national health indicators [7] contrasted with the findings of this study. The report attributed the health disparities to the economic wealth, infrastructure (availability of clean water), education, and corruption levels in the Muslim countries. There is limited information to explain oral health disparities in the present study; only the economic status of a country was considered, and it explains the disparities better than other parameters in this study. Economic disparities in oral health are continuously changing over time. Data up to the millennium suggested an increasing caries trend in 12-year-olds in developing countries compared to a decreasing trend in developed countries [18]; after the millennium, there is a global declining pattern with an attenuating effect among middle and low-income countries [19-21]. The D-component of DMFT is low in the HIC but high in the LIC, and the M-component is higher in the UMIC and HIC compared to the LIC, due to the greater number of oral health professionals, and availability and access to oral health services [22-25].

The determinants of oral health such as oral health behaviors, fluoridated toothpaste, and dental visits have contributed to the declining trend of the disease [24]. The social determinants, including the social conditions, environment, and economic conditions have broader influences on health inequalities [26,27]. Religion is one of the social stratifications that discriminate the population apart from demographic, economic, and political differences [7]. The interest in disparity by religious belief in oral health, particularly from the perspective of the Islamic religion, lies in its emphasis on hygiene practice in general and oral hygiene in particular. The teaching of Islam strongly advocates and includes oral hygiene and dental care as part of rituals; followers are strongly encouraged to gargle/rinse the mouth and clean the teeth (using the miswak stick) before each of the five daily prayers or meeting people, and after waking up from sleep [28,29]. Diligently keeping up with the practices can prevent dental plaque from accumulating throughout the day, thus lowering the risk of developing oral diseases. This notion is a key point that should always be included in the oral health messages targeting the Muslim population [28,29].

The findings of this study should be interpreted with caution for several reasons. First, the population-level analysis in ecological study design ignores the diversity of beliefs, particularly in multi-religious populations, and thus does not reflect the true effect of religion. The use of secondary data limits the number of parameters included in the analysis and issues such as mediation and confounding are not fully accounted for. This study limits the analysis to the age group 35-44 only for outcomes DMFT and periodontal disease due to inconsistent and lack of data for other age bands. The use of the DMFT index may be biased due to the inclusion of missing and filled teeth, they are measures of treated lesions that are no longer a burden to individuals. Lastly, missing data from certain countries and the sources of oral health data, which are from the published reports of national oral health surveys, journal articles and health bulletins, and personal communications [9] and may vary in the quality and availability of the most recent data, may introduce bias in the results. Nevertheless, such use of data is not uncommon and has been applied in earlier reports [7,19]. One advantage of this exploratory study is that it compared health variation between larger populations to assess the similar issues observed within a population using secondary data.

## Conclusions

This study found weak evidence for oral health disparities between non-Muslim and Muslim countries despite the significant finding showing a lower caries experience in the latter. Future investigations on the topic should consider a multi-national study and include measures of oral health behaviors and religiosity as the explanatory factor to further examine the hypothesis.

## Appendices

**Table 4:** Classification of Muslim and Non-Muslim countries used for this study (N=170)

Muslim Countries N=53 (31%)		Non-Muslim Countries N=117 (69%)	
Certainly, here is the list of countries separated by commas: Afghanistan, Albania, Algeria, Azerbaijan, Bahrain, Bangladesh, Brunei, Burkina Faso, Cameroon, Chad, Comoros, Cote d'Ivoire, Djibouti, Egypt, Gabon, The Gambia, Guinea, Guyana, Guinea, Indonesia, Iran, Iraq, Jordan, Kazakhstan, Kuwait, Kyrgyzstan, Lebanon, Libya, Malaysia, Mali, Mauritania, Morocco, Mozambique, Niger, Nigeria, Oman, Pakistan, Qatar, Saudi Arabia, Senegal, Sierra Leone, Somalia, Sudan, Syria	Tajikistan, Togo, Tunisia, Turkey, Turkmenistan, Uganda, UAE, Uzbekistan, Yemen	Antigua & Barbuda, Argentina, Armenia, Australia, Austria, Bahamas, Barbados, Bosnia Herzegovina, Belarus, Belgium, Belize, Benin, Bhutan, Bolivia, Botswana, Brazil, Bulgaria, Burundi, Cambodia, Canada, Cape Verde, Central African Republic, Chile, China, Colombia, Congo (DRC), Costa Rica, Croatia, Cuba, Cyprus, Czech Republic, Denmark, Dominica, Dominican Republic, Ecuador, El Salvador, Eritrea, Estonia, Ethiopia, Fiji, Finland, France, Georgia, Germany, Ghana, Greece, Grenada, Guatemala, Haiti, Honduras, Hungary, Iceland, India, Ireland, Israel, Italy, Jamaica, Japan, Kenya	Korea (Republic of), Lao People's Democratic Republic, Latvia, Lesotho, Liberia, Libya, Lithuania, Luxembourg, Macedonia, Madagascar, Malawi, Malta, Mauritius, Mexico, Micronesia, Moldova, Mongolia, Myanmar, Namibia, Nepal, Netherlands, New Zealand, Nicaragua, Norway, Angola, Panama, Paraguay, Peru, Philippines, Poland, Portugal, Romania, Russia, Rwanda, Serbia, Seychelles, Singapore, Slovakia, Slovenia, South Africa, Spain, Sri Lanka, Swaziland, Sweden, Switzerland, Tanzania, Thailand, Tonga, Trinidad & Tobago, Ukraine, United Kingdom, United States of America, Uruguay, Vanuatu, Venezuela, Vietnam, Zambia, Zimbabwe​​​​

**Table 5:** List of countries included in the study classified by the status of non-Muslim and Muslim countries and GNI per capita income groups (n = 170). GNI: Gross National Income

		Muslim Countries N = 53	Non-Muslim Countries N=117	
n=50		n=7	n=43	
GNI per Capita	High-Income countries	Bahrain, Brunei, Kuwait, Oman, Qatar, Saudi Arabia, UAE	Antigua & Barbuda, Australia, Austria, Bahamas, Barbados, Belgium, Canada, Croatia, Cyprus, Chile, Czech Republic, Denmark, Estonia, Finland, France, Germany, Greece, Iceland, Israel, Italy, Japan, Korea (Republic of)	Latvia, Lithuania, Luxembourg, Malta, Netherlands, New Zealand, Norway, Poland, Portugal, Russia, Ireland, Singapore, Slovenia, Slovakia, Spain, Sweden, Switzerland, Trinidad, Uruguay, United Kingdom, United States of America
	n=46	n= 14	n=32	
	Upper middle-income countries	Algeria, Albania, Azerbaijan, Gabon, Iraq, Iran, Kazakhstan, Lebanon, Libya, Jordan, Malaysia, Turkey, Tunisia, Turkmenistan	Angola, Argentina, Belarus, Botswana, Brazil, Bulgaria, Bosnia Herzegovina, China, Colombia, Costa Rica, Cuba, Dominican Republic, Dominica, Ecuador, Fiji, Hungary	Grenada, Jamaica, Montenegro, Mauritius, Macedonia, Mexico, Namibia, Panama, Peru, Romania, Seychelles, Serbia, South Africa, Thailand, Tonga, Venezuela
	n=41	n=15	n=26	
	Lower middle-income countries	Cameroon, Côte d'Ivoire, Djibouti, Egypt, Guyana, Indonesia, Morocco, Niger, Nigeria, Pakistan, Senegal, Sudan, Syria, Uzbekistan, Yemen	Armenia, Bhutan, Bolivia, Cape Verde, Congo (DRC), El Salvador, Georgia, Ghana, Guatemala, Honduras, India, Lao P.D.R, Lesotho, Micronesia, Moldova, Mongolia	Nicaragua, Papua New Guinea, Paraguay, Philippines, Sri Lanka, Swaziland, Ukraine, Vanuatu, Vietnam, Zambia
	n=33	n=17	n=16	
	Lower-income countries	Afghanistan, Bangladesh, Burkina Faso, Chad, Comoros, The Gambia, Guinea, Guinea-Bissau, Kyrgyzstan, Mali, Mauritania, Mozambique, Togo, Sierra Leone, Somalia, Tajikistan, Uganda	Burundi, Benin, Cambodia, Central African Republic, Congo (DRC), Eritrea, Estonia, Haiti, Kenya, Liberia, Madagascar, Malawi, Myanmar, Nepal, Tanzania, Zimbabwe	
